# Bis[μ-1,2-diphenyl-*N*,*N*′-bis­(di-2-pyridyl­methyl­eneamino)ethane-1,2-diimine]disilver(I) bis­(hexa­fluorido­phosphate) acetonitrile disolvate

**DOI:** 10.1107/S160053680904882X

**Published:** 2009-11-21

**Authors:** Qiaozhen Sun, Tao Xiao, Ping Chen, Xiaoqiang Gan, Lin Bao

**Affiliations:** a Department of Materials Chemistry, School of Materials Science and Engineering, Key Laboratory of Nonferrous Metals, Ministry of Education, Central South University, Changsha 410083, People’s Republic of China; b School of Chemistry and Chemical Engineering, Central South University, Changsha 410083, People’s Republic of China

## Abstract

In the centrosymmetric dinuclear title compound, [Ag_2_(C_36_H_26_N_8_)_2_](PF_6_)_2_·2C_2_H_3_N, the Ag^+^ ion is bound to four N atoms from two 1,2-diphenyl-*N*,*N*′-bis­(di-2-pyridyl­methyl­eneamino)ethane-1,2-diimine ligands in a distorted tetra­hedral geometry. The ligand adopts a twist conformation, coordinating two metal centers by three pyridyl N atoms and one imine N atom and spanning two Ag^+^ ions, resulting in the formation of a helical dimeric structure.

## Related literature

For the role of helicity in self-assembly processes in supra­molecular chemistry, see: Stefankiewicz *et al.* (2008 ▶). For examples of single- and double-stranded architectures, see: Chowdhury *et al.* (2003 ▶); Stefankiewicz *et al.* (2008 ▶). The basic features to give predicta­ble products have been established, see: Constable *et al.* (1997 ▶). We have previously reported the spontaneous resolution of silver double helicates (Sun *et al.*, 2006 ▶) and entanglemental coordination polymers of silver helicates (Sun *et al.*, 2007 ▶). For a related structure, see: He *et al.* (2000 ▶). For related literature, see: Beckmann & Brooker (2003 ▶).
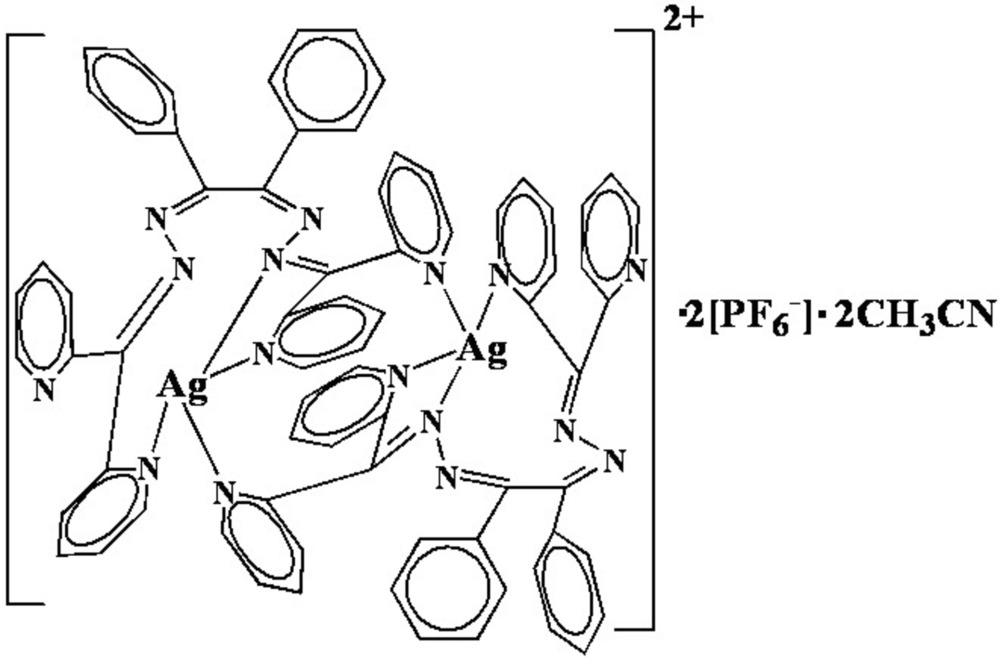


## Experimental

### 

#### Crystal data

[Ag_2_(C_36_H_26_N_8_)_2_](PF_6_)_2_·2C_2_H_3_N
*M* *_r_* = 1729.08
Triclinic, 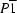

*a* = 11.595 (2) Å
*b* = 12.544 (3) Å
*c* = 13.893 (3) Å
α = 110.037 (4)°
β = 90.798 (4)°
γ = 101.192 (4)°
*V* = 1855.1 (7) Å^3^
*Z* = 1
Mo *K*α radiation
μ = 0.66 mm^−1^
*T* = 293 K
0.36 × 0.30 × 0.30 mm

#### Data collection

Bruker SMART CCD diffractometer
Absorption correction: multi-scan (*SADABS*; Bruker, 2000 ▶) *T*_min_ = 0.766, *T*_max_ = 0.813
9153 measured reflections
6299 independent reflections
3860 reflections with *I* > 2σ(*I*)
*R*_int_ = 0.058

#### Refinement

*R*[*F* ^2^ > 2σ(*F* ^2^)] = 0.062
*wR*(*F* ^2^) = 0.132
*S* = 1.00
6299 reflections
496 parameters
12 restraints
H-atom parameters constrained
Δρ_max_ = 0.94 e Å^−3^
Δρ_min_ = −0.43 e Å^−3^

### 

Data collection: *SMART* (Bruker, 2000 ▶); cell refinement: *SAINT* (Bruker, 2000 ▶); data reduction: *SAINT*; program(s) used to solve structure: *SHELXTL* (Sheldrick, 2008 ▶); program(s) used to refine structure: *SHELXTL*; molecular graphics: *SHELXTL*; software used to prepare material for publication: *SHELXTL*.

## Supplementary Material

Crystal structure: contains datablocks global, I. DOI: 10.1107/S160053680904882X/cs2124sup1.cif

Structure factors: contains datablocks I. DOI: 10.1107/S160053680904882X/cs2124Isup2.hkl

Additional supplementary materials:  crystallographic information; 3D view; checkCIF report

## Figures and Tables

**Fig. 1.**
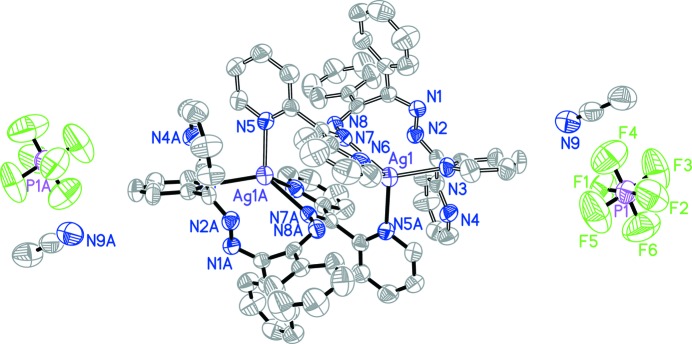
The double helical title compound showing the atom-numbering for the non-H and non-C atoms only, with the H atoms omitted for clarity, too. Displacement ellipsoids are drawn at the 50% probability level.

## supplementary crystallographic information

### Comment

Helicity continues to receive considerable attention as it allows for a greater understanding of the self-assembly processes involved in supramolecular chemistry (Stefankiewicz *et al.*, 2008). Many examples of both single- and double-stranded architectures have been reported (Chowdhury *et al.*, 2003; Stefankiewicz *et al.*, 2008). The basic features to give predictable products are established (Constable *et al.*, 1997). We have previously reported the spontaneous resolution of silver double helicates (Sun *et al.*, 2006) and entanglemental coordination polymers of silver helicates (Sun *et al.*, 2007).

The title complex is a double helical silver(I) coordination compound with the similar ligands, benzil dihydrazone-N, N'-(di-2-pyridyl-ketimine) (Fig. 1). In the dimeric double helicate, each silver(I) centre coordinates to one imine nitrogen atoms, two pyridyl N atoms from one ligand and one pyridyl N atom from symmetry related ligand, forming a distorted tetrahedral geometry. Meanwhile, we notice that unlike the structure we reported before (Sun *et al.*, 2007), each ligand coordinates with metal ions by using three pyridyl N atoms and one imine N atom, leaving one pyridyl ring of 2-pyridyl-ketimine uncoordinated. After coordination with Ag ions, the two sets of pyridine rings distorted differently with one set showing the dihedral angles of cca. 70°, and the other displaying the angles with ca. 100°. It is the twisting angle in the latter set that displaces one of the pyridyl groups unfavorable for coordination. An interesting feature of the dication is that the ligand spans both silver ions, but does not wrap around the metal-metal axis as demonstrated by the bis(pyridylmine) Schiff base ligands (He *et al.*, 2000). One of the two ligands pass above the Ag—Ag axis and the other goes beneath, with the [Ag_2_L_2_]^2+^ cation appearing more like a box than a double helix. The close distance of the silver(I) cations (Ag···Ag distance: cca. 5.02Å) seem unfavourable for helicate formation. However, it should be noted that coordination to the metal centers forces helical twisting of the ligand with the torsion angle of cca. 98° about the bond N(1)—C(7)—C(30)—N(8). Two di-2-pyridyl-ketimine moieties are found on the opposite sides of the N(1)—C(7)—C(30)—N(8) fragment, giving rise to a double helix.

### Experimental

Preparation of ligand L: An ethanolic solution (5 mL) of di-2-pyridyl-ketone (1.27 g, 8.2 mmol) was added slowly to a ethanolic solution (20 mL) of benzildihydrazone (0.98 g, 4.1 mmol) and the resulting solution was refluxed for four hours. The reaction mixture was condensed and cooled to room temperature. Upon standing overnight the resultant yellow solid was filtered off, washed with diethyl ether and dried under vacuum. Yield: 85%. Elemental analyses calcd (%): 75.8; H, 4.6; N, 19.6. Found: C, 75.6; H, 4.7; N, 19.7. 1H NMR (500 MHz, DMSO, 298 K): 8.55(d, 2H), 8.47 (d, 2H), 7.85 (t, 2H), 7.64 (d, 4H), 7.60 (t, 4H), 7.47 (t, 2H), 7.46 (d, 2H), 7.45 (d, 2H), 7.43 (d, 2H), 7.42 (t, 2H), 7.37 (t, 2H).

Preparation of the title complex: The ligand *L* (0.1 mmol, 0.057 g) and AgNO_3_ (0.15 mmol, 0.027 g) were mixed in methanol and refluxed for two hours, then added 5 mL acetonitrile solution of KPF_6_, the yellow solution was filtered and evaporated at room temperature. A few days later orange block crystals were obtained.

### Refinement

All of the non-hydrogen atoms were refined with anisotropic thermal displacement coefficients. H atoms were placed at calculated positions with C—H = 0.93–0.96Å and included in a riding-model approximation with *U*_iso_(H) = 1.2*U*_eq_(C). The order HADD was used to restraint the H atoms.

### Crystal data

**Table:**

[Ag_2_(C_36_H_26_N_8_)_2_](PF_6_)_2_·2C_2_H_3_N	*Z* = 1
*M**_r_* = 1729.08	*F*(000) = 872
Triclinic, *P*1	*D*_x_ = 1.548 Mg m^−^^3^
Hall symbol: -P 1	Mo *K*α radiation, λ = 0.71073 Å
*a* = 11.595 (2) Å	Cell parameters from 1917 reflections
*b* = 12.544 (3) Å	θ = 2.3–19.8°
*c* = 13.893 (3) Å	µ = 0.66 mm^−^^1^
α = 110.037 (4)°	*T* = 293 K
β = 90.798 (4)°	Block, yellow
γ = 101.192 (4)°	0.36 × 0.30 × 0.30 mm
*V* = 1855.1 (7) Å^3^	

### Data collection

**Table:**

Bruker SMART CCD diffractometer	6299 independent reflections
Radiation source: fine-focus sealed tube	3860 reflections with *I* > 2σ(*I*)
graphite	*R*_int_ = 0.058
φ and ω scans	θ_max_ = 25.0°, θ_min_ = 1.8°
Absorption correction: multi-scan (*SADABS*; Bruker, 2000)	*h* = −13→5
*T*_min_ = 0.766, *T*_max_ = 0.813	*k* = −14→14
9153 measured reflections	*l* = −16→16

### Refinement

**Table:**

Refinement on *F*^2^	Primary atom site location: structure-invariant direct methods
Least-squares matrix: full	Secondary atom site location: difference Fourier map
*R*[*F*^2^ > 2σ(*F*^2^)] = 0.062	Hydrogen site location: inferred from neighbouring sites
*wR*(*F*^2^) = 0.132	H-atom parameters constrained
*S* = 1.00	*w* = 1/[σ^2^(*F*_o_^2^) + (0.042*P*)^2^] where *P* = (*F*_o_^2^ + 2*F*_c_^2^)/3
6299 reflections	(Δ/σ)_max_ < 0.001
496 parameters	Δρ_max_ = 0.94 e Å^−^^3^
12 restraints	Δρ_min_ = −0.43 e Å^−^^3^

### Fractional atomic coordinates and isotropic or equivalent isotropic displacement parameters (Å^2^)

**Table:**

	*x*	*y*	*z*	*U*_iso_*/*U*_eq_	
Ag1	0.12500 (5)	0.95781 (4)	0.12566 (4)	0.0597 (2)	
N1	0.0264 (4)	0.6675 (4)	0.0581 (4)	0.0477 (12)	
N2	−0.0614 (4)	0.7111 (4)	0.1186 (4)	0.0484 (12)	
N3	0.1775 (5)	0.8631 (4)	0.2274 (4)	0.0518 (13)	
N4	−0.0751 (5)	0.8984 (5)	0.3691 (4)	0.0589 (14)	
N6	0.2588 (5)	1.0100 (4)	0.0189 (4)	0.0550 (13)	
N7	0.0420 (4)	0.8746 (4)	−0.0466 (3)	0.0439 (12)	
N8	−0.0692 (4)	0.8050 (4)	−0.0812 (3)	0.0481 (12)	
N9	0.1326 (7)	0.5150 (7)	0.4160 (6)	0.103 (2)	
C1	0.2128 (6)	0.6376 (6)	−0.0729 (5)	0.0656 (19)	
H1B	0.2364	0.6752	−0.0032	0.079*	
C2	0.2942 (6)	0.6007 (7)	−0.1391 (6)	0.089 (2)	
H2B	0.3725	0.6139	−0.1142	0.107*	
C3	0.2617 (7)	0.5442 (6)	−0.2420 (7)	0.081 (2)	
H3A	0.3173	0.5194	−0.2874	0.098*	
C4	0.1464 (7)	0.5249 (6)	−0.2767 (5)	0.070 (2)	
H4B	0.1228	0.4852	−0.3462	0.083*	
C5	0.0646 (6)	0.5635 (5)	−0.2103 (5)	0.0576 (17)	
H5A	−0.0135	0.5510	−0.2355	0.069*	
C6	0.0966 (5)	0.6205 (5)	−0.1069 (4)	0.0449 (15)	
C7	0.0102 (5)	0.6614 (4)	−0.0354 (4)	0.0418 (14)	
C8	−0.0267 (5)	0.7660 (5)	0.2139 (4)	0.0456 (15)	
C9	0.0976 (5)	0.7900 (5)	0.2574 (4)	0.0449 (14)	
C10	0.1285 (6)	0.7424 (6)	0.3259 (5)	0.0615 (18)	
H10A	0.0710	0.6931	0.3458	0.074*	
C11	0.2428 (7)	0.7662 (6)	0.3658 (5)	0.072 (2)	
H11A	0.2642	0.7314	0.4107	0.086*	
C12	0.3251 (6)	0.8425 (6)	0.3385 (5)	0.0665 (19)	
H12A	0.4030	0.8641	0.3665	0.080*	
C13	0.2877 (6)	0.8860 (6)	0.2675 (5)	0.0673 (19)	
H13A	0.3440	0.9350	0.2463	0.081*	
C14	−0.1161 (6)	0.8080 (5)	0.2831 (4)	0.0479 (15)	
C15	−0.2335 (6)	0.7572 (6)	0.2609 (5)	0.074 (2)	
H15A	−0.2594	0.6933	0.2012	0.089*	
C16	−0.3131 (7)	0.8019 (7)	0.3282 (6)	0.086 (2)	
H16A	−0.3933	0.7685	0.3146	0.104*	
C17	−0.2723 (7)	0.8959 (7)	0.4149 (6)	0.077 (2)	
H17A	−0.3236	0.9282	0.4617	0.092*	
C18	−0.1534 (7)	0.9410 (6)	0.4309 (5)	0.072 (2)	
H18A	−0.1259	1.0062	0.4894	0.086*	
C24	0.0996 (5)	0.9089 (5)	−0.1129 (4)	0.0431 (14)	
C25	0.2171 (5)	0.9865 (5)	−0.0784 (5)	0.0495 (15)	
C26	0.2823 (6)	1.0298 (6)	−0.1438 (5)	0.068 (2)	
H26A	0.2528	1.0123	−0.2113	0.082*	
C27	0.3915 (8)	1.0991 (7)	−0.1073 (7)	0.094 (3)	
H27A	0.4360	1.1297	−0.1506	0.113*	
C28	0.4358 (7)	1.1240 (7)	−0.0110 (7)	0.087 (2)	
H28A	0.5105	1.1705	0.0135	0.105*	
C29	0.3656 (6)	1.0772 (6)	0.0511 (6)	0.073 (2)	
H29A	0.3948	1.0939	0.1185	0.087*	
C30	−0.0869 (5)	0.7038 (5)	−0.0724 (4)	0.0443 (14)	
C31	−0.2037 (5)	0.6277 (5)	−0.1080 (4)	0.0472 (15)	
C32	−0.2863 (6)	0.6554 (6)	−0.1619 (5)	0.0625 (18)	
H32A	−0.2682	0.7240	−0.1757	0.075*	
C33	−0.3952 (6)	0.5833 (8)	−0.1957 (6)	0.085 (2)	
H33A	−0.4502	0.6031	−0.2322	0.102*	
C34	−0.4231 (7)	0.4823 (7)	−0.1759 (6)	0.092 (3)	
H34A	−0.4975	0.4342	−0.1971	0.110*	
C35	−0.3411 (8)	0.4533 (7)	−0.1249 (7)	0.106 (3)	
H35A	−0.3593	0.3840	−0.1124	0.127*	
C36	−0.2317 (6)	0.5243 (6)	−0.0915 (6)	0.078 (2)	
H36A	−0.1761	0.5022	−0.0575	0.094*	
C37	0.3081 (8)	0.4417 (8)	0.4581 (7)	0.117 (3)	
H37B	0.3621	0.4377	0.4058	0.140*	
H37A	0.2809	0.3657	0.4612	0.140*	
H37C	0.3474	0.4933	0.5234	0.140*	
C38	0.2105 (9)	0.4837 (7)	0.4343 (6)	0.083 (3)	
P1	0.47556 (19)	0.79693 (19)	0.62145 (17)	0.0765 (6)	
F1	0.3401 (4)	0.7611 (5)	0.5937 (3)	0.1205 (17)	
F2	0.6120 (4)	0.8324 (5)	0.6505 (5)	0.150 (2)	
F3	0.4756 (5)	0.6863 (5)	0.6438 (5)	0.171 (3)	
F4	0.4932 (5)	0.7295 (6)	0.5091 (4)	0.169 (2)	
F5	0.4802 (6)	0.9025 (6)	0.5900 (7)	0.205 (3)	
F6	0.4586 (6)	0.8673 (7)	0.7292 (5)	0.199 (3)	
N5	−0.0396 (5)	0.9091 (4)	−0.2430 (3)	0.0515 (13)	
C19	−0.0838 (6)	0.8697 (5)	−0.3419 (5)	0.0620 (18)	
H19A	−0.1478	0.8967	−0.3585	0.074*	
C20	−0.0392 (7)	0.7925 (6)	−0.4184 (5)	0.069 (2)	
H20A	−0.0736	0.7658	−0.4856	0.082*	
C21	0.0560 (7)	0.7547 (6)	−0.3959 (5)	0.071 (2)	
H21A	0.0881	0.7024	−0.4477	0.085*	
C22	0.1044 (6)	0.7941 (6)	−0.2965 (5)	0.0628 (18)	
H22A	0.1706	0.7702	−0.2797	0.075*	
C23	0.0535 (5)	0.8690 (5)	−0.2228 (4)	0.0473 (15)	

### Atomic displacement parameters (Å^2^)

**Table:**

	*U*^11^	*U*^22^	*U*^33^	*U*^12^	*U*^13^	*U*^23^
Ag1	0.0740 (4)	0.0575 (3)	0.0531 (3)	0.0185 (3)	0.0034 (3)	0.0240 (2)
N1	0.051 (3)	0.047 (3)	0.045 (3)	0.014 (3)	0.002 (3)	0.013 (2)
N2	0.049 (3)	0.056 (3)	0.044 (3)	0.015 (3)	0.009 (3)	0.021 (3)
N3	0.049 (3)	0.055 (3)	0.055 (3)	0.014 (3)	−0.007 (3)	0.021 (3)
N4	0.067 (4)	0.067 (4)	0.043 (3)	0.021 (3)	0.007 (3)	0.015 (3)
N6	0.052 (4)	0.046 (3)	0.065 (4)	0.007 (3)	0.000 (3)	0.019 (3)
N7	0.039 (3)	0.040 (3)	0.055 (3)	0.010 (2)	0.002 (3)	0.019 (2)
N8	0.049 (3)	0.051 (3)	0.050 (3)	0.016 (3)	0.005 (2)	0.023 (3)
N9	0.122 (7)	0.080 (5)	0.102 (6)	0.028 (5)	0.001 (5)	0.021 (4)
C1	0.054 (5)	0.075 (5)	0.060 (4)	0.014 (4)	0.002 (4)	0.013 (4)
C2	0.054 (5)	0.114 (7)	0.084 (6)	0.017 (5)	0.011 (5)	0.016 (5)
C3	0.068 (6)	0.080 (5)	0.097 (6)	0.028 (5)	0.033 (5)	0.024 (5)
C4	0.083 (6)	0.072 (5)	0.053 (4)	0.026 (4)	0.021 (4)	0.016 (4)
C5	0.056 (4)	0.063 (4)	0.051 (4)	0.016 (3)	0.002 (3)	0.013 (3)
C6	0.044 (4)	0.039 (3)	0.053 (4)	0.011 (3)	0.005 (3)	0.016 (3)
C7	0.042 (4)	0.034 (3)	0.043 (3)	0.000 (3)	−0.006 (3)	0.010 (3)
C8	0.057 (4)	0.044 (3)	0.043 (4)	0.013 (3)	0.005 (3)	0.023 (3)
C9	0.051 (4)	0.047 (4)	0.039 (3)	0.018 (3)	0.010 (3)	0.015 (3)
C10	0.073 (5)	0.066 (4)	0.054 (4)	0.014 (4)	−0.002 (4)	0.032 (4)
C11	0.080 (6)	0.084 (5)	0.063 (5)	0.029 (5)	−0.011 (4)	0.034 (4)
C12	0.057 (5)	0.071 (5)	0.069 (5)	0.015 (4)	−0.016 (4)	0.021 (4)
C13	0.057 (5)	0.068 (5)	0.083 (5)	0.010 (4)	0.004 (4)	0.035 (4)
C14	0.052 (4)	0.054 (4)	0.043 (4)	0.012 (3)	0.005 (3)	0.023 (3)
C15	0.066 (5)	0.064 (5)	0.076 (5)	0.006 (4)	0.015 (4)	0.007 (4)
C16	0.057 (5)	0.088 (6)	0.092 (6)	0.008 (4)	0.021 (5)	0.008 (5)
C17	0.073 (6)	0.084 (6)	0.077 (5)	0.028 (5)	0.032 (5)	0.025 (5)
C18	0.094 (6)	0.068 (5)	0.051 (4)	0.024 (5)	0.017 (4)	0.014 (4)
C24	0.049 (4)	0.039 (3)	0.049 (4)	0.017 (3)	0.009 (3)	0.021 (3)
C25	0.051 (4)	0.046 (4)	0.054 (4)	0.015 (3)	0.010 (3)	0.019 (3)
C26	0.066 (5)	0.067 (5)	0.064 (5)	−0.003 (4)	0.011 (4)	0.025 (4)
C27	0.084 (7)	0.091 (6)	0.098 (7)	−0.010 (5)	0.023 (6)	0.036 (5)
C28	0.062 (5)	0.072 (5)	0.109 (7)	−0.010 (4)	−0.003 (5)	0.021 (5)
C29	0.059 (5)	0.062 (5)	0.087 (5)	0.003 (4)	−0.010 (4)	0.019 (4)
C30	0.047 (4)	0.053 (4)	0.031 (3)	0.015 (3)	0.006 (3)	0.012 (3)
C31	0.042 (4)	0.052 (4)	0.045 (4)	0.010 (3)	0.003 (3)	0.015 (3)
C32	0.047 (4)	0.068 (4)	0.076 (5)	0.006 (4)	−0.007 (4)	0.034 (4)
C33	0.052 (5)	0.110 (7)	0.101 (6)	0.017 (5)	−0.007 (4)	0.047 (5)
C34	0.062 (6)	0.085 (6)	0.124 (7)	−0.003 (5)	−0.016 (5)	0.041 (6)
C35	0.079 (6)	0.078 (6)	0.155 (8)	−0.018 (5)	−0.034 (6)	0.053 (6)
C36	0.062 (5)	0.064 (5)	0.110 (6)	−0.004 (4)	−0.029 (4)	0.042 (4)
C37	0.108 (8)	0.110 (7)	0.117 (8)	0.032 (6)	0.011 (6)	0.017 (6)
C38	0.099 (8)	0.065 (6)	0.075 (6)	0.016 (5)	0.009 (6)	0.011 (4)
P1	0.0690 (15)	0.0782 (14)	0.0797 (15)	0.0159 (12)	−0.0054 (12)	0.0246 (12)
F1	0.063 (3)	0.180 (5)	0.110 (4)	0.010 (3)	−0.004 (3)	0.049 (4)
F2	0.074 (4)	0.120 (4)	0.229 (6)	0.016 (3)	−0.035 (4)	0.032 (4)
F3	0.136 (5)	0.168 (5)	0.264 (7)	0.013 (4)	−0.009 (5)	0.156 (6)
F4	0.162 (6)	0.213 (7)	0.112 (5)	0.029 (5)	0.035 (4)	0.037 (5)
F5	0.189 (6)	0.147 (5)	0.310 (8)	0.018 (4)	−0.041 (5)	0.133 (5)
F6	0.168 (4)	0.237 (5)	0.139 (4)	0.086 (4)	−0.004 (3)	−0.025 (4)
N5	0.067 (4)	0.049 (3)	0.042 (3)	0.013 (3)	0.005 (3)	0.019 (2)
C19	0.072 (5)	0.063 (4)	0.053 (4)	0.017 (4)	−0.004 (4)	0.022 (4)
C20	0.097 (6)	0.058 (4)	0.046 (4)	0.017 (4)	−0.002 (4)	0.013 (4)
C21	0.101 (6)	0.062 (5)	0.055 (5)	0.026 (4)	0.027 (4)	0.021 (4)
C22	0.075 (5)	0.065 (4)	0.057 (4)	0.027 (4)	0.017 (4)	0.026 (4)
C23	0.055 (4)	0.044 (3)	0.049 (4)	0.012 (3)	0.012 (3)	0.023 (3)

### Geometric parameters (Å, °)

**Table:**

Ag1—N3	2.277 (5)	C17—H17A	0.93
Ag1—N5^i^	2.291 (5)	C18—H18A	0.93
Ag1—N6	2.317 (5)	C24—C25	1.477 (8)
Ag1—N7	2.361 (5)	C24—C23	1.490 (8)
N1—C7	1.284 (6)	C25—C26	1.375 (8)
N1—N2	1.402 (6)	C26—C27	1.367 (9)
N2—C8	1.281 (6)	C26—H26A	0.93
N3—C13	1.324 (7)	C27—C28	1.336 (10)
N3—C9	1.348 (7)	C27—H27A	0.93
N4—C18	1.321 (8)	C28—C29	1.389 (10)
N4—C14	1.336 (7)	C28—H28A	0.93
N6—C29	1.328 (7)	C29—H29A	0.93
N6—C25	1.342 (7)	C30—C31	1.465 (7)
N7—C24	1.287 (6)	C31—C36	1.372 (8)
N7—N8	1.385 (6)	C31—C32	1.373 (8)
N8—C30	1.294 (7)	C32—C33	1.372 (8)
N9—C38	1.112 (10)	C32—H32A	0.93
C1—C2	1.361 (9)	C33—C34	1.366 (10)
C1—C6	1.373 (8)	C33—H33A	0.93
C1—H1B	0.93	C34—C35	1.352 (10)
C2—C3	1.370 (9)	C34—H34A	0.93
C2—H2B	0.93	C35—C36	1.371 (9)
C3—C4	1.362 (9)	C35—H35A	0.93
C3—H3A	0.93	C36—H36A	0.93
C4—C5	1.371 (8)	C37—C38	1.416 (11)
C4—H4B	0.93	C37—H37B	0.96
C5—C6	1.375 (7)	C37—H37A	0.96
C5—H5A	0.93	C37—H37C	0.96
C6—C7	1.460 (7)	P1—F6	1.493 (6)
C7—C30	1.489 (8)	P1—F3	1.522 (6)
C8—C14	1.473 (8)	P1—F5	1.522 (6)
C8—C9	1.488 (8)	P1—F4	1.537 (6)
C9—C10	1.362 (8)	P1—F1	1.552 (4)
C10—C11	1.366 (8)	P1—F2	1.565 (5)
C10—H10A	0.93	N5—C23	1.340 (7)
C11—C12	1.369 (9)	N5—C19	1.345 (7)
C11—H11A	0.93	N5—Ag1^i^	2.291 (5)
C12—C13	1.381 (8)	C19—C20	1.356 (8)
C12—H12A	0.93	C19—H19A	0.93
C13—H13A	0.93	C20—C21	1.355 (9)
C14—C15	1.368 (8)	C20—H20A	0.93
C15—C16	1.381 (9)	C21—C22	1.367 (9)
C15—H15A	0.93	C21—H21A	0.93
C16—C17	1.363 (9)	C22—C23	1.362 (8)
C16—H16A	0.93	C22—H22A	0.93
C17—C18	1.368 (9)		
			
N3—Ag1—N5^i^	99.33 (17)	N6—C25—C26	121.5 (6)
N3—Ag1—N6	119.34 (18)	N6—C25—C24	116.9 (5)
N5^i^—Ag1—N6	122.00 (16)	C26—C25—C24	121.5 (6)
N3—Ag1—N7	127.26 (16)	C27—C26—C25	118.4 (7)
N5^i^—Ag1—N7	119.22 (17)	C27—C26—H26A	120.8
N6—Ag1—N7	70.27 (17)	C25—C26—H26A	120.8
C7—N1—N2	112.5 (5)	C28—C27—C26	121.6 (8)
C8—N2—N1	114.4 (5)	C28—C27—H27A	119.2
C13—N3—C9	116.7 (5)	C26—C27—H27A	119.2
C13—N3—Ag1	120.8 (4)	C27—C28—C29	117.1 (7)
C9—N3—Ag1	122.2 (4)	C27—C28—H28A	121.5
C18—N4—C14	117.2 (6)	C29—C28—H28A	121.5
C29—N6—C25	118.1 (6)	N6—C29—C28	123.3 (7)
C29—N6—Ag1	124.5 (5)	N6—C29—H29A	118.3
C25—N6—Ag1	116.4 (4)	C28—C29—H29A	118.3
C24—N7—N8	116.3 (5)	N8—C30—C31	117.7 (5)
C24—N7—Ag1	116.6 (4)	N8—C30—C7	121.5 (5)
N8—N7—Ag1	126.5 (3)	C31—C30—C7	120.5 (5)
C30—N8—N7	116.2 (5)	C36—C31—C32	118.2 (6)
C2—C1—C6	121.4 (6)	C36—C31—C30	120.5 (6)
C2—C1—H1B	119.3	C32—C31—C30	121.3 (6)
C6—C1—H1B	119.3	C33—C32—C31	120.9 (7)
C1—C2—C3	120.5 (7)	C33—C32—H32A	119.5
C1—C2—H2B	119.7	C31—C32—H32A	119.5
C3—C2—H2B	119.7	C34—C33—C32	120.1 (7)
C4—C3—C2	118.7 (7)	C34—C33—H33A	119.9
C4—C3—H3A	120.6	C32—C33—H33A	119.9
C2—C3—H3A	120.6	C35—C34—C33	119.2 (8)
C3—C4—C5	120.7 (7)	C35—C34—H34A	120.4
C3—C4—H4B	119.6	C33—C34—H34A	120.4
C5—C4—H4B	119.6	C34—C35—C36	121.2 (8)
C4—C5—C6	120.8 (6)	C34—C35—H35A	119.4
C4—C5—H5A	119.6	C36—C35—H35A	119.4
C6—C5—H5A	119.6	C35—C36—C31	120.3 (7)
C1—C6—C5	117.7 (6)	C35—C36—H36A	119.8
C1—C6—C7	121.0 (5)	C31—C36—H36A	119.8
C5—C6—C7	121.3 (6)	C38—C37—H37B	109.5
N1—C7—C6	118.6 (5)	C38—C37—H37A	109.5
N1—C7—C30	123.0 (5)	H37B—C37—H37A	109.5
C6—C7—C30	118.1 (5)	C38—C37—H37C	109.5
N2—C8—C14	117.2 (5)	H37B—C37—H37C	109.5
N2—C8—C9	123.8 (5)	H37A—C37—H37C	109.5
C14—C8—C9	118.9 (5)	N9—C38—C37	178.7 (11)
N3—C9—C10	121.7 (6)	F6—P1—F3	94.9 (4)
N3—C9—C8	116.8 (5)	F6—P1—F5	90.1 (4)
C10—C9—C8	121.5 (6)	F3—P1—F5	174.8 (5)
C9—C10—C11	120.8 (6)	F6—P1—F4	177.3 (5)
C9—C10—H10A	119.6	F3—P1—F4	87.8 (4)
C11—C10—H10A	119.6	F5—P1—F4	87.2 (4)
C10—C11—C12	118.6 (6)	F6—P1—F1	90.8 (3)
C10—C11—H11A	120.7	F3—P1—F1	91.2 (3)
C12—C11—H11A	120.7	F5—P1—F1	90.1 (3)
C11—C12—C13	117.3 (6)	F4—P1—F1	89.2 (3)
C11—C12—H12A	121.3	F6—P1—F2	88.8 (3)
C13—C12—H12A	121.3	F3—P1—F2	88.2 (3)
N3—C13—C12	124.8 (6)	F5—P1—F2	90.5 (3)
N3—C13—H13A	117.6	F4—P1—F2	91.2 (4)
C12—C13—H13A	117.6	F1—P1—F2	179.3 (4)
N4—C14—C15	122.2 (6)	C23—N5—C19	116.5 (5)
N4—C14—C8	115.7 (6)	C23—N5—Ag1^i^	125.8 (4)
C15—C14—C8	122.0 (6)	C19—N5—Ag1^i^	117.7 (4)
C14—C15—C16	119.2 (6)	N5—C19—C20	123.0 (6)
C14—C15—H15A	120.4	N5—C19—H19A	118.5
C16—C15—H15A	120.4	C20—C19—H19A	118.5
C17—C16—C15	118.9 (7)	C21—C20—C19	119.2 (6)
C17—C16—H16A	120.6	C21—C20—H20A	120.4
C15—C16—H16A	120.6	C19—C20—H20A	120.4
C16—C17—C18	117.9 (7)	C20—C21—C22	119.5 (7)
C16—C17—H17A	121.0	C20—C21—H21A	120.3
C18—C17—H17A	121.0	C22—C21—H21A	120.3
N4—C18—C17	124.4 (7)	C23—C22—C21	118.4 (7)
N4—C18—H18A	117.8	C23—C22—H22A	120.8
C17—C18—H18A	117.8	C21—C22—H22A	120.8
N7—C24—C25	118.1 (5)	N5—C23—C22	123.4 (6)
N7—C24—C23	122.1 (5)	N5—C23—C24	116.5 (5)
C25—C24—C23	119.7 (5)	C22—C23—C24	120.1 (6)

Symmetry codes: (i) −*x*, −*y*+2, −*z*.
